# Processing-Enhanced β-Phase Formation in BaTiO_3_/PVDF Composite Fibers with High Electroactive Phase Content

**DOI:** 10.3390/nano16110664

**Published:** 2026-05-25

**Authors:** Marouene Ben Ouali, Anik Das, Chayma Ben Harrath, Xu Lei, Rony Mia

**Affiliations:** 1 School of Textile Science and Engineering, Tiangong University, Tianjin 300387, China; 2 College of Textiles and Clothing, Qingdao University, Qingdao 266071, China; 3 Department of General Medicine, Saveetha Medical College and Hospital, Saveetha Institute of Medical and Technical Sciences, Chennai 602105, Tamil Nadu, India; 4 Department of Textile Engineering, Chemistry and Science, Wilson College of Textiles, North Carolina State University, Raleigh, NC 27606, USA

**Keywords:** barium titanate, nanoparticles, polyvinylidene fluoride, piezoelectric material, melt spinning, composite fiber

## Abstract

Flexible piezoelectric fibers are promising materials for next-generation wearable and flexible electronic devices due to their lightweight structure, mechanical flexibility, and electromechanical response. In this study, BaTiO_3_/PVDF composite fibers were prepared by melt spinning under an electrostatic field, followed by thermal drawing to enhance the electroactive phase content. The effects of BaTiO_3_ loading, draw ratio, thermal stretching ratio, stretching rate, and electric field strength on the crystalline structure of the fibers were systematically investigated. Fourier transform infrared spectroscopy, X-ray diffraction, differential scanning calorimetry, and electron microscopy were used to evaluate phase evolution, crystallinity, and filler distribution. The results showed that the processing conditions significantly influenced the transformation of PVDF from the α-phase to the electroactive β-phase. The optimized fibers were obtained at 1 wt.% BaTiO_3_, a thermal stretching ratio of 5, a stretching rate of 40 mm/min, and an electric field strength of 18 kV, resulting in a crystallinity of 61.3% and a β-phase content of 95.5%. The enhanced structural characteristics indicate the strong potential of the developed composite fibers for flexible electroactive applications, though direct electromechanical characterization is required for device integration.

## 1. Introduction

Polyvinylidene fluoride (PVDF) is a semi-crystalline polymer with excellent mechanical properties, chemical resistance, high elasticity, thermoelectric and piezoelectric properties [1]. The main crystalline forms of PVDF are non-polar α phase and polar β phase. The weight content of β phase is closely related to piezoelectric properties. In order to obtain excellent piezoelectric properties, the β phase content in PVDF should be increased as much as possible [2].

PVDF melt-spun fibers have the characteristics of small diameter, low weight, and high flexibility. They can be used as micro transducers with single-fiber structures, or they can be woven into planar structures for large-area sensors or actuators [3,4,5]. However, the crystal form of PVDF fibers obtained by ordinary melt spinning is mostly α crystal when naturally cooled and crystallized. Therefore, in order to optimize the β content in PVDF, it is necessary to transform the α crystal into the desired β crystal through methods such as hot stretching and electric field polarization [6,7,8]. In addition to stretching and polarization treatment, nanofillers are also an important method to enhance the β phase content in PVDF. Kai et al. prepared PVDF and multi-walled carbon nanotube (MWCNT)-based nanocomposites with different surface functionalizations by melt mixing on a small scale [9]. They found that the incorporation of MWCNTs enabled the β phase to be directly cooled from the melt in PVDF, and MWCNTs helped the formation of the β phase. Chen et al. incorporated polyethylene glycol-grafted graphene into PVDF to form a novel PEG-graphene/PVDF solution blending method to prepare composite materials [10]. The presence of PEG-graphene effectively enhanced the electroactive crystal content of graphene, increasing PVDF from 24.6% in pure PVDF to 90.5% in PEG-graphene (15 wt.%)/PVDF. It also had a higher dielectric constant of 53.3. BaTiO_3_ piezoelectric ceramics possess high piezoelectric conversion efficiency [11]. Adding BTO nanowires during spinning can cause the nanowires to distribute along the fiber orientation, promoting the nucleation of the β phase in PVDF [12,13], potentially leading to single fibers with higher β crystal content and piezoelectric constants. However, there are currently few reports on the preparation of PVDF using BTO nanowire doping. Beyond its role as a piezoelectric ceramic in polymer composites, BaTiO_3_ has recently attracted growing interest across diverse application fields. For instance, recent studies have explored the photocatalytic activity of doped BaTiO_3_, such as rhodium-doped BaTiO_3_ for enhanced performance under visible light [14]. BaTiO_3_/CuO nanocomposites have also been investigated for their photocatalytic efficiency under both visible and UV irradiation [15]. In parallel, synthesis strategies continue to evolve, with sol–gel routes being tailored to produce highly tetragonal BaTiO_3_ nanoparticles through controlled aging time and calcination temperature [16]. These emerging directions underscore the versatility of BaTiO_3_ beyond conventional piezoelectrics. In the context of PVDF-based composites, the incorporation of BaTiO_3_ nanoparticles serves a dual purpose: they act as heterogeneous nucleating agents promoting the electroactive β-phase of PVDF, and they contribute to the overall piezoelectric response of the composite. This makes BaTiO_3_/PVDF systems particularly attractive for flexible electronics, energy harvesting, and sensing applications.

Although BaTiO_3_-filled PVDF composites and mechanically drawn PVDF fibers have been reported previously, most studies have focused on films, electrospun mats, printed inks, or conventionally compounded composites [2,3]. In contrast, the present work focuses on BaTiO_3_/PVDF monofilament fibers prepared through a combined solvent-assisted BaTiO_3_ pre-dispersion, electrostatic-field-assisted melt-spinning, and thermal-drawing strategy. This approach was designed to improve BaTiO_3_ distribution before melt processing, promote interfacial nucleation of the electroactive β-phase, and further enhance molecular orientation through post-drawing. The novelty of this work lies in clarifying the coupled effects of BaTiO_3_ loading, melt-spinning draw ratio, thermal-stretching ratio, stretching rate, and electric-field strength on β-phase development in melt-spun BaTiO_3_/PVDF monofilaments. This study therefore provides both processing optimization and mechanistic insight into β-phase formation in flexible composite fibers with high electroactive phase content.

## 2. Materials and Methods

### 2.1. Experimental Materials

Polyvinylidene fluoride (PVDF) granules (model FR916) were supplied by Shanghai Sanai Fu New Materials Co., Ltd., Shanghai, China. The material had a melting point of 172 °C, a density of 1.77 g/cm^3^, a melt flow index of 24 g/10 min (230 °C, 5.0 kg), and a moisture content of 0.01 wt.%. N,N-dimethylformamide (DMF, analytical grade) was purchased from Tianjin Kemei Chemical Reagent Co., Ltd., Tianjin, China. Barium titanate (BTO) nanoparticles with an average particle size of 100 nm were obtained from Shanghai Aladdin Reagent Co., Ltd., Shanghai, China. 

A plunger-type melt-spinning machine (AT226 Series, Anytester (Hefei) Co., Ltd., Hefei, Anhui, China) equipped with a spinneret of 0.6 mm diameter was used for fiber preparation. The winding speed of the system was 2 m/min. An electrostatic generator (DW750) manufactured by Tianjin Dongwen High Voltage Power Supply Co., Ltd., Tianjin, China, was used to provide the electrostatic field during spinning.

### 2.2. Preparation of BTO/PVDF Composite Fibers

#### 2.2.1. Preparation of BTO/PVDF Nascent Fibers

**Figure 1:**
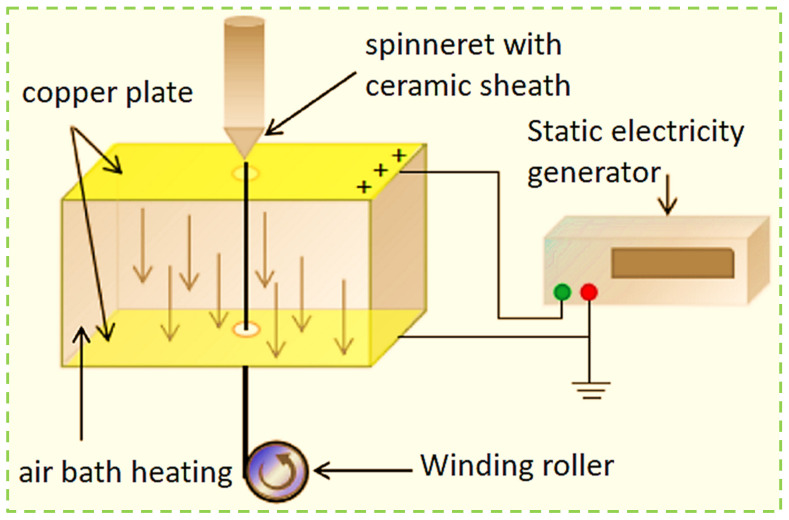
PVDF melt spinning process under electrostatic assistance.

BTO nanoparticles were ultrasonically dispersed in DMF for 30 min, followed by the addition of dried PVDF spun particles in different proportions. The mixture was stirred at 60 °C for 2 h until completely dissolved. After evaporating the solvent, the prepared BaTiO_3_/PVDF mixture was melt-sliced to obtain BaTiO_3_/PVDF mixtures with BTO contents of 0.25 wt.%, 0.5 wt.%, 0.75 wt.%, 1 wt.%, and 1.25 wt.%. The BaTiO_3_/PVDF mixture was sliced and fed into a plunger-type melt spinning apparatus, as shown in Figure 1, and uniformly heated to 185 °C. The spinneret diameter was 0.6 mm. By adjusting the winding rate, PVDF fiber monofilaments with draw ratios of 1.5, 2.5, 3.5, 4.5, and 5.5 were spun. This solvent-assisted pre-dispersion step was used to reduce BaTiO_3_ particle agglomeration before melt spinning. Unlike direct dry blending, the preliminary ultrasonic dispersion in DMF allowed the nanoparticles to be distributed within the PVDF matrix prior to melt processing. During the spinning process, an electrostatic field of 18 kV/12 cm was applied to the hot air heating zone for all fiber samples, unless otherwise specified. The electrostatic field was applied immediately after fiber extrusion, with the electrode positioned perpendicular to the fiber drawing direction at a fixed distance of 12 cm. The temperature of the hot-air heating zone was maintained at 185 °C, and the nascent BTO/PVDF fiber monofilaments were collected.

#### 2.2.2. Thermal Drawing of Fibers

On an Instron 5669 universal tensile testing machine, Instron, Norwood, MA, USA, equipped with a constant temperature chamber, multiple fiber samples were fixed with paper frames and heated uniformly to 90 °C. The nascent fibers were subjected to hot stretching treatment, with the original fibers stretched 3–6 times at speeds of 10 mm/min, 20 mm/min, 40 mm/min, and 60 mm/min, respectively. After that, the samples were allowed to stand for annealing for 10 min and then removed from the constant temperature chamber at room temperature. After stretching, the fibers were allowed to anneal at room temperature for 10 min before removal from the chamber.

### 2.3. Analytical Testing

#### 2.3.1. Field Emission Electron Microscopy (SEM)

The microstructure of the fiber surface was analyzed using a Gemini SEM500 thermal field emission scanning electron microscope manufactured by ZEISS GmbH, Oberkochen, Germany, with an accelerating voltage of 10 kV.

#### 2.3.2. Infrared Spectrum of Fibers (FTIR)

The crystal structure of the composite fiber was tested using a Nicolet IS50 Fourier transform infrared spectrometer manufactured by Thermo Fisher Scientific, Waltham, MA, USA, with a scanning range of 4000–400 cm^−1^. The spectra were collected and processed using OMNIC software, version 9.0. The weight content of the β-crystal form was calculated using the Gregorio method based on the infrared spectral curves [12].

(1) $$F_{\left(\beta \right)}=\frac{A_{\beta }}{(K_{\beta }/K_{\alpha })A_{\alpha }+A_{\beta }}=\frac{A_{\beta }}{1.26A_{\alpha }+A_{\beta }}$$

where F(β) is the content of the β crystal form in the fiber crystalline phase; K_β_ and K_α_ are the molar area coefficients, which are 7.7 × 10^4^ cm^2^/mol and 6.1 × 10^4^ cm^2^/mol, respectively; and A_α_ and A_β_ are the characteristic absorption intensities of the α and β crystal form bands.

#### 2.3.3. X-Ray Diffraction (XRD)

The fiber crystal structure was tested using a D8 DISCOVER X-ray diffractometer manufactured by BRUKER AXS SE, Karlsruhe, Germany, with a wavelength λ of 0.15418 nm. The fibers were arranged vertically and parallel at the intersection of the X-ray beams, and the scanning range 2θ was 5–40°. The XRD curves were calculated using the following formulas: I_β_/I_α_ (%) and X_β_ (%) content [11]:

(2) $$\frac{I_{\beta }}{I_{\alpha }}=\frac{A_{\beta }}{A_{\alpha }}$$

(3) $$X_{\beta }=\frac{A_{\beta }}{A_{\alpha }+A_{\beta }}$$

where A_α_ and A_β_ are the crystallization peak areas corresponding to the α and β crystal planes, respectively; I_β_/I_α_ is the intensity ratio of β to α crystal forms; and X_β_ is the crystallinity of the β crystal.

#### 2.3.4. Differential Scanning Calorimeter (DSC)

The thermal properties of the fiber were tested using a DSC200FS differential scanning calorimeter from Netzsch GmbH, Oberkochen, Germany. The temperature was measured under a nitrogen atmosphere with a programmed heating rate of 10 °C and a temperature range of 20–230 °C. The formula for calculating the crystallinity of the fiber using the DSC curve is shown in Equation (4) [12,17].

(4) $$X_{C}=\frac{{\Delta H}_{m}}{{\Delta H}_{0}}\times 100\%$$

where ΔH_m_ is the melting enthalpy of the sample (J/g); ΔH_0_ is the melting enthalpy of the PVDF resin sample in its complete or uncompleted form (104.7 J/g); and X_C_ is the crystallinity of the sample.

It should be noted that while DSC allows for the estimation of total crystallinity, the assignment of melting peaks to specific crystalline phases (α, β, γ) requires correlation with complementary techniques such as FTIR and XRD, as peak positions can vary with thermal and mechanical history [18].

Three different quantities are reported in this study to characterize the crystalline structure of the fibers:

- F(β) from FTIR: Fraction of the β-phase within the crystalline regions only (i.e., β/(α + β)). This quantity ranges from 0 to 100% and indicates the relative dominance of the β-phase among the crystalline phases, but does not provide information on the total amount of crystallinity.
- X_c_ from DSC: Total crystallinity of the fiber, defined as the fraction of the polymer that is crystalline (α + β) relative to the total polymer mass. This is calculated using X_c_ = ΔH_m_/ΔH_0_, where ΔH_0_ = 104.7 J/g for 100% crystalline PVDF.
- X_β_ from XRD: Crystallinity of the β-phase only, defined as the fraction of the total polymer mass that is in the β-crystalline form. In principle, X_β_ = X_c_ × F(β). However, because XRD and DSC probe different aspects of crystalline order, small discrepancies may exist between measured and calculated values.

It should be noted that while DSC is used here to calculate total crystallinity (X_c_), phase identification is based primarily on FTIR and XRD, as melting peaks of different PVDF crystalline phases (α, β, γ) can overlap [7,19].

## 3. Results and Discussion

### 3.1. Nascent BaTiO_3_/PVDF Fibers with Different Draw Ratios

**Figure 2:**
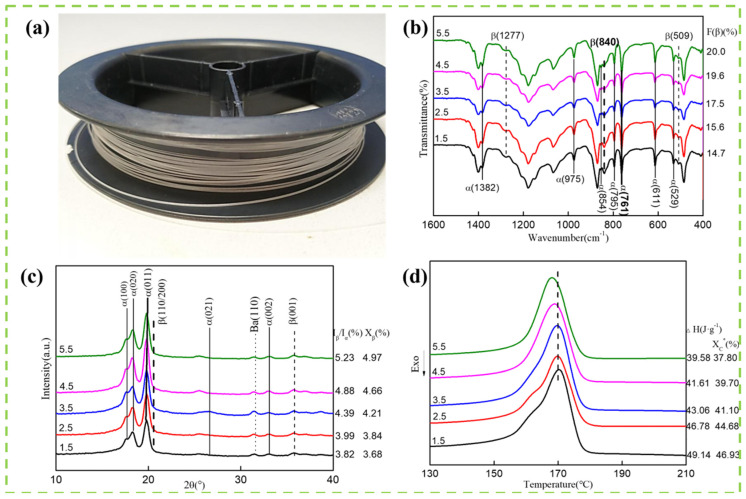
(**a**) Physical image of nascent BaTiO_3_/PVDF fibers; (**b**) FTIR spectra; (**c**) XRD patterns; (**d**) DSC diagrams of nascent BaTiO_3_/PVDF fibers at different draw ratios.

Figure 2a shows the physical image of nascent BaTiO_3_/PVDF fibers, with the monofilament having a uniform diameter and continuous length. Figure 2b shows the FTIR spectra of BaTiO_3_ content at 1 wt.% and electric field strength at 18 kV under different draw ratios. The absorption peaks of the inorganic filler (i.e., BTO) are very weak and undetectable compared to the main PVDF peaks [13]. Therefore, there is no significant difference between the spectra of unfilled PVDF and BaTiO_3_/PVDF composites. Figure 2b shows α characteristic peaks at 1382, 975, 854, 795, 761, 611, and 529 cm^−1^, with a significant α characteristic peak at 761 cm^−1^, and only weak β characteristic peaks at 1277, 840, and 509 cm^−1^. This indicates that the PVDF fiber crystals at different draw ratios are mainly α crystals, with a small amount of β crystals. From F(β)% in Figure 2b, it can be seen that the crystallinity increases slowly with an increase in the draw ratio. The small amount of β crystal form is due to the fact that some PVDF is pulled by the spinneret during extrusion, and under stress, a small part of the fiber changes from α to β crystal form. This is because when the draw ratio is low, the PVDF has an irregularly coiled macromolecular structure. When the spinneret is drawn, the macromolecules inside the PVDF are arranged in an orderly manner, the coiled macromolecules unfold, and with the partial chain forging movement, the crystal form changes. Since the spinning winding rate has a slow effect on the improvement of the fiber β crystal form, a 5-times draw ratio is selected to prepare nascent fibers, which is beneficial for providing a higher draw ratio and better elongation at break during post-drawing [20].

Figure 2c shows the XRD patterns of nascent BaTiO_3_/PVDF fibers with a BaTiO_3_ content of 1 wt.% and an electrostatic field strength of 18 kV under different draw ratios. Diffraction peaks appear at 17.7°, 18.4°, 19.9°, 20.6°, 26.6°, 31.5°, 33.1°, and 36.6°, corresponding to crystal planes α(100), α(020), α(011), β(110/200), α(021), Ba(110), α(002), and β(001). Obvious α diffraction peaks are observed at 2θ of 17.7°, 18.4°, and 19.9°, while almost no β diffraction peak is observed at 20.6°, with only a weak β diffraction peak at 36.6°. This indicates that the BaTiO_3_/PVDF fibers under different draw ratios mainly exist in the α crystal form, consistent with the FTIR results. As shown in Figure 2c, the effect of increasing draw ratio on fiber β crystal content is not significant. To make the fibers more resistant to stretching during post-stretching, a stretch ratio of 5 is selected.

Figure 2d shows the DSC diagrams of nascent BaTiO_3_/PVDF fibers under different draw ratios; two peaks, at 163 °C and 170 °C, appear in the figure. In the DSC thermograms (Figure 2d), two melting peaks are observed at approximately 163 °C and 170 °C. Based on the FTIR and XRD results showing that the as-spun fibers are predominantly α-phase (Figure 2b,c), the major peak at 170 °C is attributed to the α-phase. The smaller peak at 163 °C is attributed to the β-phase, consistent with previous reports on melt-spun PVDF fibers [19,21]. As the draw ratio increases, the melting peak shifts toward lower temperatures, indicating an increase in β-phase content. It should be noted that while DSC allows estimation of total crystallinity, the assignment of melting peaks to specific crystalline phases (α, β, γ) requires correlation with complementary techniques such as FTIR and XRD, as peak positions can vary with thermal and mechanical history [18], and the peak corresponding to 170 °C is the α characteristic peak. The characteristic peak at 170 °C is significantly higher than that at 163 °C, indicating that the internal crystals of the fiber mainly exist in the α crystal form. As the draw ratio increases, the melting temperature gradually moves to a lower temperature, indicating that the increase in draw ratio helps to form the β crystal form of the fiber and increases the crystallinity of the fiber.

### 3.2. Composite Fibers After Thermal Stretching

**Figure 3:**
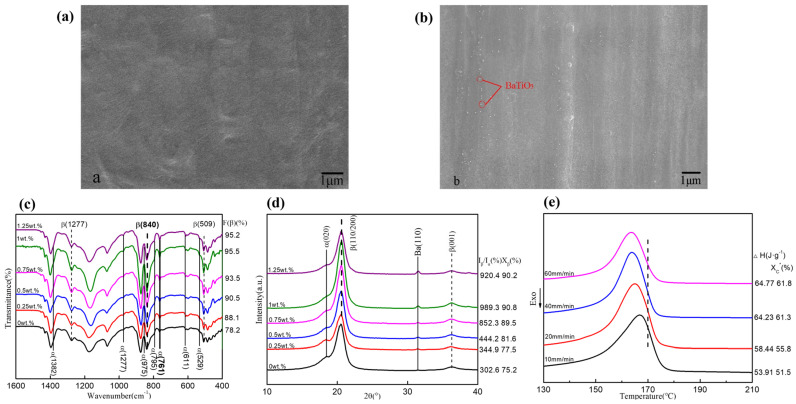
SEM images of the fiber radial direction: (**a**) PVDF monofilament; (**b**) PVDF fiber with 1 wt.% BaTiO_3_; (**c**) FTIR spectra; (**d**) XRD patterns; (**e**) DSC diagrams of BaTiO_3_/PVDF fibers with different BaTiO_3_ contents.

**Figure 4:**
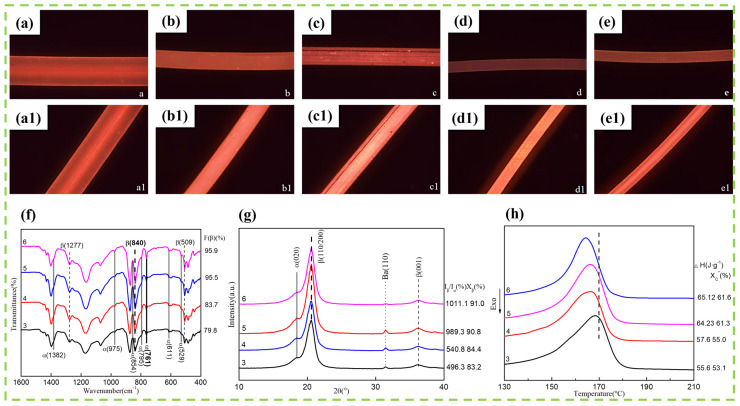
(**a**–**e1**) POM diagrams of BaTiO_3_/PVDF fibers at different post-stretch ratios ((**a**–**e**) represent 0, 3, 4, 5, and 6 times the thermal stretch ratio, respectively; (**a1**–**e1**) represent the stage rotated by 45°); (**f**) FTIR spectra; (**g**) XRD patterns; (**h**) DSC thermograms of BaTiO_3_/PVDF fibers at different thermal stretching ratios.

**Figure 5:**
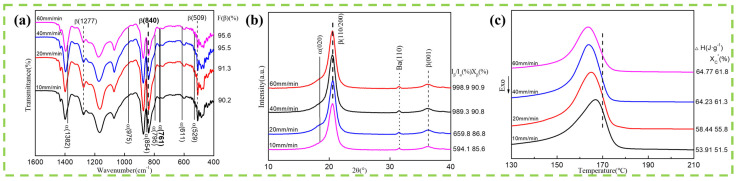
(**a**) FTIR spectra; (**b**) XRD patterns; (**c**) DSC thermograms of BaTiO_3_/PVDF fibers at different thermal stretching rates.

**Figure 6:**
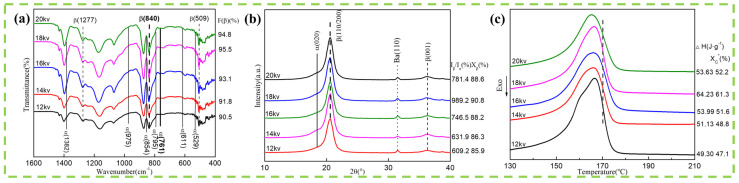
(**a**) FTIR spectra; (**b**) XRD patterns; (**c**) DSC thermograms of BaTiO_3_/PVDF fibers under different electric field intensities.

Table 1 summarizes the optimal processing conditions for each parameter and the corresponding β-phase content (F(β)). Although a thermal stretching ratio of 6 gives a slightly higher F(β) (95.9%), ratio 5 was selected as optimal due to better mechanical integrity (see Section 3.2.2). For the complete evolution of F(β) across the full range of each parameter, please refer to Figure 2b, Figure 3c, Figure 4f, Figure 5a and Figure 6a.

#### 3.2.1. BaTiO_3_/PVDF Fibers with Different BaTiO_3_ Contents

To characterize the microstructure of BaTiO_3_/PVDF fibers, SEM was performed on the fiber surface, as shown in Figure 3a,b. The pure PVDF fiber surface (Figure 3a) was smooth and free of particles. The composite fiber with 1 wt.% BaTiO_3_ showed BaTiO_3_ particles on the surface without large-scale aggregation, indicating that the BaTiO_3_ particles were relatively uniformly dispersed (Figure 3b). Figure 3c shows the FTIR spectra of BaTiO_3_/PVDF fibers with different BaTiO_3_ contents under a 5× draw ratio, 5× hot stretching, a hot stretching rate of 40 mm/min, and an electric field strength of 18 kV. As shown in Figure 3c, with the continuous increase in BaTiO_3_ content, the α characteristic peak at 761 cm^−1^ weakens, while the β characteristic peak at 840 cm^−1^ strengthens with increasing content. When BaTiO_3_ content is 0 wt.%, F(β) is 78.2%, significantly lower than the F(β) value of the doped composite fiber. With increasing content, when the content reaches 1 wt.%, F(β) reaches its maximum value of 95.5%. The content continued to increase, but F(β) did not increase significantly. This indicates that BaTiO_3_ can act as a nucleating agent to promote the crystallization activity of PVDF during the crystallization process. The polymer chains are arranged on the surface of BT particles in the BaTiO_3_ conformation. Therefore, in the β phase, a strong interaction is generated between CH_2_ and BT in the PVDF chain [19]. Figure 3d shows the XRD patterns of BaTiO_3_/PVDF fibers with different BaTiO_3_ contents. As can be seen from Figure 3d, with an increase in BaTiO_3_ content, the α(020) diffraction peak at 18.4° gradually weakens, the α diffraction peak disappears at 19.9°, and the β diffraction peak gradually strengthens, indicating that the fiber mainly contains the β crystal form. When 2θ is 20.4°, the β(110/200) diffraction peak gradually increases, and the β diffraction peak is strongest when the BaTiO_3_ content reaches 1 wt.%. Corresponding to the data in Figure 3d, an increase in BaTiO_3_ content gradually increases X_β_ (%). When the content is 1 wt.%, the I_β_/I_α_ (%) value is 989.3, and X_β_ (%) reaches a maximum of 90.8%. This indicates that an increase in BaTiO_3_ content enhances crystallization activity and promotes the crystallization of the β crystal form, consistent with the results of FTIR analysis in Figure 3c. Therefore, a BaTiO_3_ content of 1 wt.% is optimal.

Figure 3e shows the DSC curves of BaTiO_3_/PVDF fibers with different BaTiO_3_ contents. It can be observed from Figure 3e that when the BaTiO_3_ content is 0.25 wt.%, the melting temperature is slightly below 170 °C, indicating the presence of a small amount of β-crystal form in the fibers. When the BaTiO_3_ content reaches 0.5 wt.%, a distinct α-characteristic peak appears with significantly higher intensity than the α melting peak, suggesting an enhanced β-crystal content in the fibers. At a BaTiO_3_ content of 0.75 wt.%, the α melting peak further shifts toward the β melting peak, with the β-crystal form becoming dominant. When the BaTiO_3_ content reaches 1 wt.%, the α melting peak almost disappears, while the β melting peak exhibits a larger area and a sharp shape. When the BaTiO_3_ content is 0 wt.%, the crystallinity of the fibers is 51.2%, which is lower than the crystallinity of 53% at a BaTiO_3_ content of 0.25 wt.%. As the BaTiO_3_ content increases, the fiber crystallinity also increases, reaching its highest value at a content of 1 wt.%. This indicates that the increased BaTiO_3_ content makes the fiber crystallization more standardized, which helps in the transformation from α to β crystal form.

The enhancement of β-phase formation at low BaTiO_3_ loading can be attributed to a combination of heterogeneous nucleation and interfacial dipole interaction. BaTiO_3_ particles provide nucleation sites that restrict random PVDF chain packing and favor chain conformations associated with the polar β-phase. At the same time, interfacial interactions between the ceramic surface and PVDF segments can facilitate the alignment of –CH_2_– and –CF_2_– dipoles, promoting the transition from the non-polar α-phase to the electroactive β-phase. However, when the BaTiO_3_ content exceeds the optimum level, additional particles may no longer provide effective nucleation sites because local aggregation can reduce the available interfacial area. This explains why the β-phase content increased up to 1 wt.% BaTiO_3_ but did not improve substantially at higher loading.

It should be noted that F(β) (FTIR) represents the fraction of the β-phase within the crystalline regions only, whereas X_c_ (DSC) represents the total crystallinity (all phases), and X_β_ (XRD) represents the β-phase crystallinity relative to the total polymer mass. Ideally, X_β_ ≈ X_c_ × F(β). In our optimized sample, X_c_ = 61.3% and F(β) = 95.5%, which would predict X_β_ ≈ 58.5%. The higher X_β_ value (90.8%) obtained from XRD suggests that different assumptions or baseline corrections were used in the XRD calculation [11]. We have therefore reported the values as obtained from each method without forcing mathematical consistency.

For comparison, fibers with 1.25 wt.% BaTiO_3_ were also prepared. Table 2 summarizes the results.

The slight decrease in all three parameters at 1.25 wt.% compared to 1 wt.% suggests that excess BaTiO_3_ particles agglomerate, reducing the effective interfacial area for heterogeneous nucleation. This explains why 1 wt.% is the optimal loading.

It should be noted that while the decrease in F(β), X_β_, and X_c_ at 1.25 wt.% BaTiO_3_ (Table 2) indirectly suggests particle agglomeration, direct imaging (SEM, TEM, or EDS) of the 1.25 wt.% sample was not performed. Such characterization is planned for future work to directly confirm the aggregation mechanism.

To contextualize our results, Table 3 compares the β-phase content obtained in this work with previously reported values for PVDF-based fibers and composites.

As shown in the previous Table 3, the β-phase content achieved in this study (95.5%) is significantly higher than previously reported values for melt-spun PVDF fibers (70–75%) and BaTiO_3_/PVDF composites (80–85%). This demonstrates that the combined use of solvent-assisted pre-dispersion, electrostatic-field-assisted melt spinning, and controlled thermal drawing is an effective strategy for promoting α-to-β phase transformation.

#### 3.2.2. BaTiO_3_/PVDF Fibers at Different Thermal Stretching Ratios

Figure 4a–e1 shows the polarizing microscope (POM) images of BaTiO_3_/PVDF fibers at thermal stretching ratios of 0, 3, 4, 5, and 6, with a BaTiO_3_ weight content of 1 wt.% in each sample. During testing, the polarizer and the stage were orthogonal in their vibration directions, in an extinction position, with the field of view in darkness. Figure 4a–e were taken in this state. The stage was rotated 45° to a diagonal position, providing the brightest field of view; Figure 4a1–e1 were taken in this state. As BaTiO_3_/PVDF fibers are stretched uniaxially in the opposite direction, the fiber length increases and the diameter decreases. The molecular chains are affected by external force and point in the direction of that force. In this direction, the macroscopic properties of the polymer are different from those in other directions, and the material is anisotropic [7]. In terms of optical properties, the orientation of the polymer leads to the occurrence of birefringence. Therefore, the degree of orientation of BaTiO_3_/PVDF fibers can be judged by the difference between the extinction position and the diagonal position under different thermal stretching ratios using a polarized light microscope. Figure 4a,a1 show that the brightness of the nascent PVDF fibers at the diagonal position and the extinction position is almost the same, with no essential difference. This indicates that the nascent fibers are mainly isotropic when they have not undergone thermal stretching treatment. That is, the orientation of the macromolecules in the fibers is not good at this time, and the arrangement is relatively disordered. Figure 4b,b1 show the composite fibers stretched 3 times. It can be seen that the brightness in Figure 4b,b1 begins to improve. The brightness at the diagonal position is stronger than that at the extinction position. The fiber orientation gradually increases and the anisotropy gradually increases. The field of view at the extinction position in Figure 4c becomes darker and darker, while the field of view at the diagonal position in Figure 4c1 becomes brighter and brighter. The fiber orientation becomes stronger and stronger. When the thermal stretching ratio reaches 5, the darkest area is the extinction position, and the brightest area is the diagonal position, indicating that the BaTiO_3_/PVDF fiber has the highest orientation and anisotropy at this point. Further increasing the thermal stretching ratio reduces the orientation of the composite fiber.

Figure 4a–e1 shows the polarizing microscope (POM) images of BaTiO_3_/PVDF fibers at thermal stretching ratios of 0, 3, 4, 5, and 6, with a BaTiO_3_ weight content of 1 wt.% in each sample. During testing, the polarizer and the stage were orthogonal in their vibration directions, in an extinction position, with the field of view in darkness. Figure 4a–e were taken in this state. The stage was rotated 45° to a diagonal position, providing the brightest field of view; Figure 4a1–e1 were taken in this state. As BaTiO_3_/PVDF fibers are stretched uniaxially in the opposite direction, the fiber length increases and the diameter decreases. The molecular chains are affected by external force and point in the direction of that force. In this direction, the macroscopic properties of the polymer are different from those in other directions, and the material is anisotropic [7]. In terms of optical properties, the orientation of the polymer leads to the occurrence of birefringence. Therefore, the degree of orientation of BaTiO_3_/PVDF fibers can be judged by the difference between the extinction position and the diagonal position under different thermal stretching ratios using a polarized light microscope. Figure 4(a,a1) show that the brightness of the nascent PVDF fibers at the diagonal position and the extinction position is almost the same, with no essential difference. This indicates that the nascent fibers are mainly isotropic when they have not undergone thermal stretching treatment. That is, the orientation of the macromolecules in the fibers is not good at this time, and the arrangement is relatively disordered. Figure 4b,b1 show the composite fibers stretched 3 times. It can be seen that the brightness in Figure 4b,b1 begins to improve. The brightness at the diagonal position is stronger than that at the extinction position. The fiber orientation gradually increases and the anisotropy gradually increases. The field of view at the extinction position in Figure 4c becomes darker and darker, while the field of view at the diagonal position in Figure 4c1 becomes brighter and brighter. The fiber orientation becomes stronger and stronger. When the thermal stretching ratio reaches 5, the darkest area is the extinction position, and the brightest area is the diagonal position, indicating that the BaTiO_3_/PVDF fiber has the highest orientation and anisotropy at this point. Further increasing the thermal stretching ratio reduces the orientation of the composite fiber.

Thermal stretching contributes to β-phase formation by extending and aligning PVDF molecular chains along the fiber axis. During stretching, the chain conformation changes from the less ordered α-phase arrangement toward the all-trans conformation characteristic of the β-phase. The applied tensile stress also improves chain packing and dipole orientation, which is consistent with the observed increase in β-phase peak intensity in FTIR and XRD. The plateau beyond a stretching ratio of 5 suggests that most transformable α-phase domains have already been converted, while further stretching mainly increases the risk of mechanical damage rather than producing substantial additional β-phase formation.

Figure 4f shows the FTIR spectra of BaTiO_3_/PVDF fibers containing 1 wt.% BaTiO_3_, prepared at a fixed hot-stretching speed of 40 mm/min and an electric field strength of 18 kV, under different hot-stretching ratios. The hot-stretching ratio refers to the degree to which the fibers are stretched relative to their original length. As the drawing ratio increases, the β-phase characteristic peak at 840 cm^−1^ intensifies sharply compared to the as-spun fiber, while the α-phase peak at 761 cm^−1^ weakens dramatically and nearly disappears. This indicates that stress-induced crystallization occurs, accompanied by segmental motion of the polymer chains, leading to a pronounced α-to-β crystal phase transformation. During stretching, the α-phase spherulites are elongated along the fiber axis, and the molecular chains become more extended and aligned until they transform into the β-phase structure [19,21]. The β-crystal content increases significantly with the drawing ratio, reaching 95.9% at a ratio of 6. However, when the hot-stretching ratio exceeds 5, the rate of increase in β-crystal content slows down. Considering that a higher hot-stretching ratio also reduces fiber fracture strength, a hot-stretching ratio of 5 is determined to be optimal. Figure 4g shows the XRD patterns of BaTiO_3_/PVDF fibers obtained at different hot-stretching ratios. The results further confirm that the crystal structure evolves progressively with increasing fiber extension. The β-crystalline phase becomes dominant, while the α (011) diffraction peak at 19.9° is almost overlapped by the β (110/200) peak. In addition, the α (020) diffraction peak gradually decreases as the stretching ratio increases. Both I_β_/I_α_ (%) and X_β_ (%) increase continuously with increasing hot-stretching ratio. At a stretching ratio of 6, I_β_/I_α_ (%) and X_β_ (%) reach maximum values of 1011.1 and 91%, respectively. Nevertheless, when the stretching ratio exceeds 5, the increase in X_β_ slows down, which is consistent with the FTIR results. This behavior can be attributed to the tensile force applied along the fiber axis during stretching. As the stretching ratio increases, the molecular chains undergo greater segmental motion and conformational rearrangement under stress, which promotes the deformation of the α-crystal form and its transformation into the β-crystal form. The α-to-β phase transition during stretching is associated with molecular reorientation and the dipole alignment of CF_2_ groups perpendicular to the fiber direction [22].

Figure 4h shows the DSC thermograms of BaTiO_3_/PVDF fibers at different hot-stretching ratios. With an increasing stretching ratio, the melting peak shifts slightly toward lower temperatures, indicating that higher fiber extension is favorable for β-phase formation. Moreover, the Xc* data show that the crystallinity of the fibers gradually increases with an increasing hot-stretching ratio.

In summary, the FTIR, XRD, and DSC results consistently demonstrate that increasing the hot-stretching ratio up to 5 promotes β-phase formation and crystallinity, while further stretching offers diminishing returns and may compromise mechanical integrity.

#### 3.2.3. BaTiO_3_/PVDF Fibers with Different Stretching Rates

Figure 5a shows the FTIR spectra of BaTiO_3_/PVDF fibers containing 1 wt.% BaTiO_3_, obtained at a fixed hot-stretching ratio of 5 and an electric field strength of 18 kV, under different hot-stretching rates. Unlike the previous section, which examined the effect of stretching extent, this section focuses on the effect of how rapidly the fibers are stretched. As the hot-stretching rate increases, the β-phase content gradually rises, indicating that a faster stretching process promotes crystal-phase transition and enhances the alignment of macromolecular chains along the fiber axis. In Figure 5a, F(β) increases progressively with increasing stretching rate, and the most favorable hot-stretching rate is 40 mm/min. When the rate exceeds 40 mm/min, the increase in F(β) becomes marginal, suggesting that further acceleration provides little additional benefit. The XRD results further support this trend. As shown in Figure 5b, the α(020) diffraction peak gradually weakens and nearly disappears as the hot-stretching rate increases, while the other characteristic peaks remain nearly unchanged. This indicates that increasing the stretching rate promotes the reduction of the α-phase and the development of the β-phase. Correspondingly, the intensity ratio I_β_/I_α_ (%) and the β-phase crystallinity X_β_ (%) increase with increasing hot-stretching rate. This behavior can be attributed to the higher internal tensile stress generated when the fiber is stretched more rapidly, which intensifies segmental motion and molecular orientation, thereby facilitating crystal transformation [21,23]. These results are in good agreement with the FTIR analysis. However, when the stretching rate becomes excessively high, the fiber becomes more susceptible to breakage. Therefore, 40 mm/min is considered the optimal hot-stretching rate. Figure 5c presents the DSC thermograms of BaTiO_3_/PVDF fibers prepared at different hot-stretching rates. As the stretching rate increases, the melting peak shifts toward lower temperatures, indicating an increase in β-phase content. In addition, the X_c_* value increases continuously with increasing hot-stretching rate, demonstrating that faster hot stretching contributes to a gradual improvement in fiber crystallinity.

#### 3.2.4. BaTiO_3_/PVDF Fibers Under Different Electric Field Strengths

As shown in Figure 6a, F(β) gradually increases with increasing electric field strength. This is because the fiber is not fully cured at high temperatures due to polarization. When an electrostatic field is applied along the fiber direction, the dipoles within the fiber orient themselves along the electric field direction, which in turn orients them along the fiber direction. This enhances molecular mobility, changes the molecular conformation, and facilitates crystal transformation. The greater the electric field strength, the more pronounced the dipole orientation and the higher the β crystal content. As shown in Figure 6b, the diffraction peak at α (020) gradually disappears with increasing electric field strength, while I_β_/I_α_ (%) and X_β_ (%) gradually increase, reaching maximum values of 989.2 and 90.8 respectively at 18 kV. This is consistent with FTIR analysis. The optimal electric field strength is 18 kV. Figure 6c shows that with increasing electric field strength, the β-crystal content of the fiber increases, and the crystallinity X_c_* also continuously improves. This indicates that increasing the electric field strength promotes fiber crystallization and the transformation of β-crystals.

The applied electrostatic field assists dipole orientation while the extruded fiber remains thermally mobile. Under the field, polar –CF_2_– dipoles tend to orient more favorably, reducing the energetic barrier for β-phase stabilization. Therefore, the electric field works synergistically with BaTiO_3_ nucleation and thermal drawing: BaTiO_3_ promotes heterogeneous nucleation, drawing aligns the chains, and the electric field assists dipole orientation. The optimum at 18 kV indicates that sufficient field strength is required for dipole alignment, whereas further increases may not produce additional β-phase growth because the available transformable domains become limited.

The applied voltage of 18 kV over an electrode distance of 12 cm corresponds to a nominal electric field of 1.5 kV/cm (E = V/d = 18 kV/12 cm). This field strength is within the typical poling range for PVDF (0.5–2 kV/cm) reported in the literature [19,24] and was sufficient to orient the –CF_2_ dipoles, as confirmed by the high β-phase content (95.5%) at 18 kV and the plateau observed beyond 18 kV (Figure 6a).

It should be noted that for the fiber diameters in this study (approximately 50–150 µm after thermal drawing), the applied electric field of 18 kV/12 cm was sufficient for effective dipole orientation. For significantly larger diameter fibers (millimeter scale), a higher field would likely be required to maintain the same poling efficiency, as the electric field strength E = V/d scales inversely with diameter.

## 4. Conclusions

BaTiO_3_/PVDF composite fibers were successfully prepared by melt spinning followed by hot drawing, and the effects of BTO content, draw ratio, hot-stretching ratio, hot-stretching rate, and electric field strength on the crystal structure of the fibers were systematically investigated. The results showed that the incorporation of BTO promoted the formation of the β-phase in PVDF fibers, indicating that BTO acted as an effective nucleating agent. In addition, increasing the draw ratio, hot-stretching ratio, hot-stretching rate, and electric field strength further enhanced the β-phase content and crystallinity of the composite fibers. Under the optimal processing conditions, namely a BTO content of 1 wt.%, a draw ratio of 5, a hot-stretching ratio of 5, a hot-stretching rate of 40 mm/min, and an electric field strength of 18 kV, the fiber crystallinity reached 61.3% and the β-phase content reached 95.5%. These results demonstrate that the combined use of BTO nanoparticles and appropriate post-drawing conditions is an effective strategy for preparing PVDF-based fibers with high β-phase content, suggesting strong potential for electroactive applications. Future work will include direct characterization of the electromechanical response (e.g., output voltage, d_33_ coefficient). The main contribution of this work is the demonstration of a combined solvent-assisted BaTiO_3_ pre-dispersion, electrostatic-field-assisted melt spinning, and thermal drawing approach for producing β-phase-rich BaTiO_3_/PVDF monofilament fibers. The observed β-phase enhancement is attributed to the synergistic effects of BaTiO_3_-induced heterogeneous nucleation, improved filler dispersion at low loading, drawing-induced molecular-chain alignment, and electric-field-assisted dipole orientation. It should be noted that the present study focuses on structural optimization; direct piezoelectric measurements (e.g., output voltage under cyclic loading, dielectric constant, and piezoelectric coefficient d_33_) are not included but are planned for subsequent investigations.

## Figures and Tables

**Table 1:** Optimal processing conditions for each parameter.

Parameter	Optimal Value	F(β) at Optimal Condition
BaTiO_3_ content (wt.%)	1.0	95.5%
Thermal stretching ratio	5	95.5%
Thermal stretching rate (mm/min)	40	95.5%
Electric field strength (kV)	18	95.5%

**Table 2:** Comparison between different wt.% of BaTiO_3_.

BaTiO_3_ (wt.%)	F(β)%	X_β_ (%)	X_c_ (%)
1.0	95.5	90.8	61.3
1.25	95.2	90.2	59.7

**Table 3:** Comparison of the present study with previous work.

Reference	Processing Method	Filler	β-Phase Content
Lund and Hagström [3]	Melt spinning	None	~70%
Defebvin et al. [2]	Solvent casting	BaTiO_3_ (5 wt.%)	~85%
Ding et al. [11]	Solution casting	BaTiO_3_ (5 wt.%)	~80%
Talbourdet et al. [7]	Melt spinning	None	~75%
This work	Melt spinning + electrostatic field + thermal drawing	BaTiO_3_ (1 wt.%)	95.5%

## Data Availability

The original contributions presented in this study are included in the article. Further inquiries can be directed to the corresponding authors.
